# Immune System and Psychological State of Pregnant Women during COVID-19 Pandemic: Are Micronutrients Able to Support Pregnancy?

**DOI:** 10.3390/nu14122534

**Published:** 2022-06-18

**Authors:** Alessio Alesci, Simona Pergolizzi, Angelo Fumia, Anthea Miller, Caterina Cernigliaro, Maria Zaccone, Vanessa Salamone, Enza Mastrantonio, Sebastiano Gangemi, Giovanni Pioggia, Nicola Cicero

**Affiliations:** 1 Department of Chemical, Biological, Pharmaceutical and Environmental Sciences, University of Messina, 98166 Messina, Italy; spergolizzi@unime.it; 2 Department of Clinical and Experimental Medicine, University of Messina, 98147 Messina, Italy; sebastiano.gangemi@unime.it; 3 Department of Veterinary Sciences, University of Messina, 98168 Messina, Italy; anthea.miller@icloud.com; 4 Azienda Sanitaria Provinciale 5 Messina, 98124 Messina, Italy; katycernigliaro@icloud.com (C.C.); mzaccone@live.it (M.Z.); vanessa.salamone3@hotmail.com (V.S.); mastrenz.a@hotmail.it (E.M.); 5 Institute for Biomedical Research and Innovation (IRIB), National Research Council of Italy (CNR), 98125 Messina, Italy; giovanni.pioggia@cnr.it; 6 Department of Biomedical and Dental Science and Morphofunctional Imaging, University of Messina, 98125 Messina, Italy

**Keywords:** immune system, pregnancy, stress, COVID-19, vitamins, micronutrients

## Abstract

The immune system is highly dynamic and susceptible to many alterations throughout pregnancy. Since December 2019, a pandemic caused by coronavirus disease 19 (COVID-19) has swept the globe. To contain the spread of COVID-19, immediate measures such as quarantine and isolation were implemented. These containment measures have contributed to exacerbate situations of anxiety and stress, especially in pregnant women, who are already particularly anxious about their condition. Alterations in the psychological state of pregnant women are related to alterations in the immune system, which is more vulnerable under stress. COVID-19 could therefore find fertile soil in these individuals and risk more severe forms. Normally a controlled dietary regimen is followed during pregnancy, but the use of particular vitamins and micronutrients can help counteract depressive-anxiety states and stress, can improve the immune system, and provide an additional weapon in the defense against COVID-19 to bring the pregnancy to fruition. This review aims to gather data on the impact of COVID-19 on the immune system and psychological condition of pregnant women and to assess whether some micronutrients can improve their psychophysical symptoms.

## 1. Introduction

Pregnancy is a unique and particular immune state [1]. The maternal immune system must tolerate the paternal antigens present in the fetus and at the same time maintain its ability to defend against possible pathogens. Physiological pregnancy consists of three stages: first pro-inflammatory phase (I trimester), anti-inflammatory phase (II and III trimesters), and second pro-inflammatory phase (late III trimester and childbirth) [2]. During pregnancy, the immune system and the body itself are highly dynamic and subject to several changes [2].

The uterus expands by increasing pressure in the abdominal cavity, lifting the diaphragm. The sub-costal angle of the rib cage increases and the ribs expand outward, decreasing chest compliance by about 35–40% [3,4]. This natural mechanism explains the “physiological dyspnea” of pregnant women and can complicate a possible diagnosis of lung disease. In addition, the residual functional capacity (RFC) is reduced, leading to compensatory respiratory alkalemia [5], shifting the oxygen balance toward the fetus. This respiratory state, following a viral infection, could easily decompensate, leading to further lung complications [6].

During pregnancy, estrogens and progesterone also play a key role in modulating the immune response [7]. Estrogens implement the response of T helper 2 lymphocytes (Th2) and humoral immunity [8]. These hormones are crucial in modulating the interaction between mother and fetus and in blocking the natural killer cells (NKs) that could be activated against fetal cells [7], showing that these mechanisms are crucial for a successful pregnancy. Plasma volume increases, lowering hemoglobin concentration. Hemoglobin is an oxygen carrier, so its diminishing can cause a decrease in the oxygen content of the blood. Anemia in pregnant women is found in about 38% [9]. In addition, many pregnant women suffer from iron deficiency, further aggravating the anemic condition. Iron is also involved in immunosurveillance mechanisms, modulating the proliferation and activation of immune cells and cytokines [4,10], phylogenetically conserved non-antigen-specific polypeptide mediators that serve as communication signals between immune system cells, organs, and tissues [11]. Moreover, during pregnancy, psychological state and dietary balance play an important role in the modulation of the immune system and the success of pregnancy [12].

The pandemic initiated by coronavirus disease 19 (COVID-19) has spread throughout the world since December 2019, when it was first reported in Wuhan, China. This disease had a globally disastrous effect, causing the death of more than 6 million people by May 2022 [13]. The main symptoms caused by severe acute respiratory syndrome coronavirus 2 (SARS-CoV-2) are fever, cough, vomit, diarrhea, anosmia, dysgeusia, and, in worst cases, interstitial bilateral pneumonia, multiple organ dysfunction, and consequences that can lead to death [14,15,16,17]. To limit the diffusion of COVID-19, urgent measures such as quarantine and isolation were adopted [18].

All these events increased individual psychological stress, exacerbating it in pregnant women, resulting in uncontrolled and compensatory food search, such as snacks and sugar-rich foods [19], and lack of exercise [20]. Stress and depression in quarantine have contributed to the practice of unhealthy and unregulated diets [21]. This has led to metabolic problems, such as increased body fat and insulin resistance, and immune problems, with an increased screening of pro-inflammatory cytokines [22].

Psychological stress is known to be one of the causes of hyperglycemia, which in pregnant women can lead to gestational diabetes [23]. Malnutrition is related to impaired immune function and chronic inflammatory states [24,25]. Adhesion to the Mediterranean Diet before, during, and after pregnancy, could reduce the risks related to the fetus and improve mood and immune system. In this sense, the use of natural and nutraceutical supplements could represent the keystone to support the successful outcome of pregnancy, strengthen the immune system, and counteract stress and depression [26,27,28,29,30].

Recently the use of nutraceuticals and natural compounds as an alternative or integrative therapy is receiving growing interest [31,32]. Polyphenols, such as anthocyanins, and micronutrients, such as minerals and vitamins, are becoming regular supplements in the diet. They possess a wide range of therapeutic biological effects, such as cholesterol-lowering [33], antioxidant [34,35,36,37], hypoglycemic, neuroprotective [38], antitumoral [39], antiviral, antibacterial, immunostimulant [31,39,40], and antidepressant [30]. Recently the role of vitamins as a therapeutic complement for SARS-CoV-2 infection has been investigated, with encouraging results [41].

This review aims to provide useful information on how COVID-19 affected the immune system and psychological state of pregnant women during the pandemic and to assess whether some micronutrients can limit or even improve the psycho-physical state of pregnant women and their clinical profile.

## 2. Immune System of Pregnant Women during COVID-19

Pregnant women represent a vulnerable segment of the population at high risk of serious COVID-19 infection, and they are 1.5 times more subject to be admitted to the Intensive Care Unit (ICU) than non-pregnant women [42]. However, the presence of infection was not related to adverse pregnancy outcomes [43]. Vertical transmission of infection from mother to fetus is extremely limited [44,45], although several studies have shown the presence of the virus in the placenta [46,47]. To date, studies have not shown frequent verticalization events of infection; however, anti-SARS-CoV-2 immunoglobulin G (IgG) has been found in newborns of COVID-19 positive women [48,49]. Subsequently, by laboratory techniques (RT-qPCR), a possible mother-to-fetal transmissibility rate of 11% within the first 48 h of life was estimated [50]. SARS-CoV-2 receptors in fetal organs and the maternal-fetal interface have also been demonstrated [51,52]. During a physiological pregnancy, the immune system changes supporting the tolerance of the fetus and protecting the body from external pathogens. The ratio between regulatory T cells (Treg) and T helper lymphocytes 17 (Th17) is the key to maintain this mechanism [46]. The Treg cells and the cytokines they produce play a key role in the preservation and development of the fetus [53], while the Th17 cells are responsible for the defense against pathogens. This balance during pregnancy shifts in favor of Treg cells, which increase both at the systemic level and on the mother-fetus side [54]. An uncontrolled increase of Th17 cells, and thus a decompensation in this delicate balance, can lead to rejection of the fetus [55] and other severe complications such as abortion, preeclampsia, and preterm birth [46].

Infection with SARS-CoV-2 causes an imbalance of this ratio in favor of an increased level of Th17 cells, leading to a state of uncontrolled systemic phlogosis, risking very serious complications for the outcome of pregnancy [46]. In a normal pregnancy, T and B-cell-mediated immunity decrease, especially during the third trimester [56], with high levels of interleukin 4 and 10 (IL-4, IL-10) and interferon γ (IFN γ) [57]. Several studies have shown that infection with the influenza A virus during pregnancy has enhanced the phlogistic response, exacerbating cytokine levels [7,58]. Pregnant women are more exposed to respiratory viral infections, such as influenza A, SARS-CoV-1, and MERS-CoV-1 [59,60]. The Centre for Disease Control and Prevention (CDC) has shown that COVID-19 positive pregnant patients do not have a higher risk of death than their non-pregnant counterparts [61,62]. In a study by Chen et al., it was noted that the cytokine response was suppressed in pregnant women with SARS-CoV-2 [15,63]. Immunological and hormonal variables have been credited for the low mortality risk. A key role in limiting COVID-19 induced damage is played by estrogens and progesterone.

Estrogen receptors are commonly expressed in white blood cells. CD4+ T cells express high levels of estrogen α receptors, while B cells express higher levels of β receptors [64]. Monocytes and CD8+ cells express lower but almost similar levels of both receptors [64]. In particular, estradiol (E2) shows an immunomodulatory and anti-inflammatory effect [65,66], inhibiting the production of IL-6, IL-1β, and Tumor Necrosis Factor α (TNFα), important in the formation of cytokine storm induced by SARS-CoV-2. It also exhibits inhibitory action on CCL2 chemokine, blocking the migration of monocytes and neutrophils at the sites of flogosis [65]. E2 decreases the release of IL-17 by Th17 and promotes Treg cells, also enhancing fetal tolerance [65]. Estrogens cooperate in limiting COVID-19 pathogenesis by interfering with pro-inflammatory cytokines. In a mouse model, female mice showed lower viral concentrations, with less infiltration of monocyte macrophages and neutrophils with lower levels of IL-6, IL-1β, TNFα, and CCL2, showing less lung damage and lower mortality (20%) than males (80%) [67]. The surgical removal of the ovaries led to equal percentages between male and female mice, demonstrating the protective effect of estrogens.

**Figure 1:**
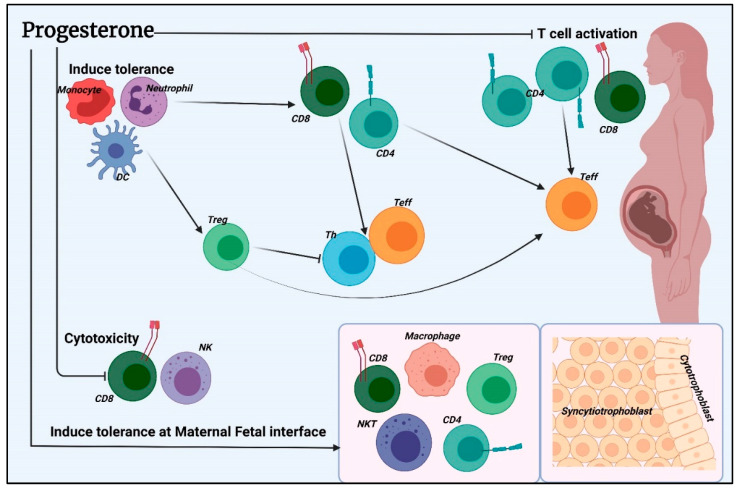
Summary of progesterone immunomodulatory mechanism. P4 directly influences T cell activation and differentiation by modulating TCR signal transduction or indirectly by generating tolerant antigen-presenting cells (APCs) such as dendritic cells (DC) that inhibit T cell activation during TCR interaction. P4 can also inhibit cellular cytotoxicity. P4 can promote placental tissue growth and invasion at the maternal-fetal interface by inducing immune-tolerant phenotypes of macrophages, natural killer (NK), and T regulatory (Treg) cells, as well as exhausting activated CD4 and CD8 T cells that have interacted with placental-derived fetal-paternal antigens. Chemoattractant molecules produced on placental tissue help these tolerant leukocytes migrate to maternal-fetal contact. Created with BioRender.com.

Progesterone (P4) also exhibits immunomodulatory and anti-inflammatory activity and is secreted in large quantities during pregnancy. P4 receptors are normally expressed by several immune cells, such as macrophages, dendritic cells, lymphocytes, mast cells, and eosinophils [65]. It has an inhibitory effect on cytokines IL-1β and IL-12 and shifts the immune balance towards Treg cells, promoting and supporting tolerance towards the fetus [65,68] (Figure 1).

Concentrations of P4 similar to those present in the lutein phase conferred protection against influenza A-induced pneumonia in mice, decreasing the inflammatory state, improving lung function, and stimulating cell proliferation [69]. Scientific evidence shows that P4 also has an antiviral activity in VeroE6 cells infected with SARS-CoV-2 [70]. P4 and human chorionic gonadotropin may act by inhibiting Th1, containing cytokine storm, and limiting the evolution of severe forms of COVID-19 [71].

Pregnant women during the first and third trimesters show a pro-inflammatory state that could facilitate the cytokine storm induced by SARS-CoV-2, causing more severe pathological conditions in these patients [72]. The transition during pregnancy from a pro-inflammatory state to an anti-inflammatory phase may depend on estrogens and progesterone, to better assist the fetal tolerance mechanism [73]. Pregnant women appear to be relatively protected from serious COVID-19-induced outcomes [74,75]. During pregnancy, there are several adaptive changes in the respiratory and immune systems that increase morbidity in pregnant women [76]. In the third trimester of pregnancy, there is an intensification in circulating phagocytes and dendritic cells, immune cells that are evolutionarily conserved with the role of antigen-presenting cells (APC) [77,78,79], capable of producing IFN type I, essential for the antiviral response. In addition, a decrease in T, B, and NK cells is evident [80]. A possible release of viral RNA in cells activates Toll-like receptors (TLRs), a group of phylogenetically conserved receptors [81,82,83,84,85,86,87], which induce the release of pro-inflammatory cytokines. These activate epithelial cells that recruit innate immune cells (NK cells and neutrophils). In addition, dendritic cells exhibit antigen by activating mediated CD4+ and CD8+ T responses [88]. Alterations due to COVID-19 infections can lead to preeclampsia, preterm birth, and emergency cesarean surgery [89]. The immune profile of pregnant and non-pregnant women is almost similar, differing only in lower blood white cell counts in pregnant women COVID-19 positive [15,90].

One of the consequences of COVID-19 infection is lymphopenia, accompanied by a major release of cytokines, called a “cytokine storm”, which leads to a very severe form of bilateral pneumonia, with extensive widespread tissue damage [91]. The susceptibility to this infection varies from individual to individual and is closely related to immune status. Immune dysfunction can therefore play an important role in the evolution of the disease [92,93]. Patients healed from COVID-19 showed a decrease in immature B cells, CD4+ memory T cells, and CD8+ T cells [94], while they had high plasma cell levels [95]. These data were compared with those of pregnant women who were COVID-19 positive after healing. Decreasing Th17 cells, belonging to memory cells and specific NK-virus cells (CD3-CD56+NKP46+) were found. NKP46+ is an NK cell activation receptor involved in interactions against pathogens [91]. Lymphopenia occurs during normal pregnancy. However, in pregnant women with SARS-CoV-2, no major changes were found in this particular condition. Marked infiltration of CD68+ macrophages has been observed in the placenta, which had slight hypoxic characteristics, but it did not cause any particular problem during childbirth [91].

## 3. Psychological Profile of Pregnant Women during COVID-19

Pregnancy involves a series of changes both hormonal and psychological, altering the emotional and sensitive state, due to the uncertainty, unpredictability, and novelty of the situation [96,97]. This stage of life brings women into a particularly vulnerable psychopathological state. Between 10% and 16% of pregnant women suffer from depression [98], while about 8% suffer from anxiety [99], with serious effects on fetal and maternal health, such as preterm birth, low birth weight, postpartum depression, and hypothalamic-pituitary-adrenal dysregulation in newborns [100,101].

The COVID-19 pandemic, especially in its early stages, has had a very strong psychological impact, with an increasing prevalence and severity of mental health problems in the general population [102], especially in pregnant women, due to the implementation of health precautions such as quarantine, wearing masks, and social distancing [103]. Immunological functions, physiological changes, and susceptibility to infection in pregnant women lead to increased experiences of stress, anxiety, and depression. A cross-sectional study conducted in China between February and March 2020 examined a total of 560 pregnant women with a mean age of 25.8 years, who were given a questionnaire with questions about attitudes and mental health toward COVID-19. The psychological impact caused by this disease has been significantly associated with the trimester of pregnancy in which these women were found. There was more evidence of psychological distress during the second trimester of pregnancy, which also harmed health (work stress, stress at home, apprehension, and helplessness), although pregnant women paid more attention to their mental health and performed relaxation activities [104,105]. A similar result emerged in Italian pregnant women from a recent systematic review on the same subject [106].

During the first year of the pandemic, symptoms of depression, anxiety, and post-traumatic stress were detected. Among the major risk factors for the development of these psychopathologies are increased concern for the health of the other person, previous mental disorders, and lack of social support. Women who have had critical antenatal and postnatal experiences during the dissemination of COVID-19 tend to focus on the other, decentralizing, ignoring, and replacing personal needs to take care of the newborn [107].

A study by Ravaldi et al. (2020), also conducted in Italy, focused on expectations and concerns regarding childbirth of pregnant women during the pandemic. While in the pre-pandemic period birth was accompanied by joy and excitement, after the spread of COVID-19, birth was associated with fear and sadness in more than half of the women interviewed. Other reasons related to fear were: loneliness, anguish, incapacity, and constraint [108]. In addition, isolation and separation from the family in the vicinity of childbirth have had a traumatic effect on COVID-19 positive women who, feeling lonely in the hospital setting, lacked social support, which is crucial in the perinatal period [109].

In the United States, a recent study (2021) found that 36.4% of pregnant women had clinically significant levels of depression, higher than the generally accepted prevalence of 20% [110]. One in five women also had clinical thresholds for generalized anxiety, and one in ten reported significant levels of post-traumatic stress disorder (PTSD). Women with a pre-existing diagnosis of mental health problems were also 1.5 to almost 4 times more likely to develop symptoms above the clinical threshold for anxiety, depression, and PTSD [111].

It is therefore necessary to provide psychological and social support for pregnant women during this period of uncertainty to avoid and minimize serious and invalidating perinatal and postnatal consequences for parents and the newborn [112]. Individuals with less social support experience greater mental health problems, especially at high levels of negative cognitive assessment. It is therefore considered necessary to raise awareness among health professionals of the increased risk to which pregnant women are exposed to mental health problems and, at the same time, to draw up clear guidelines to provide the best possible care for these patients, even in the event of a pandemic, using e.g., electronic interventions, as suggested by several studies [113,114]. Psychological therapies of choice include Cognitive Behavioral Therapy (CBT) and mindfulness-based approaches: both manage to reduce anxiety and depression in perinatal women through a structured treatment plan that trains people to internalize their attention to regularize their emotions and attention [112,115].

## 4. Micronutrients Helping Pregnancy

A correct intake of micronutrients can help the success of a physiological pregnancy and enforce the immune system of the mother, through diet or with additional dietary supplements [116]. These micronutrients, such as iron (Fe), zinc (Zn), selenium (Se), and vitamins play a key role in strengthening innate and specific immunity and preventing possible risks of pregnancy [117], while a deficiency of these nutrients can induce alterations in the immune system and make the organism most susceptible to infections [118,119]. A summary of the micronutrients herein described, with recommended intake and further suggestions can be found in Table 1.

**Iron (Fe).** Iron is an essential micronutrient and strengthens several immune functions. It is involved in the proliferation and differentiation of T cells and is a fundamental component of some enzymes linked to immune cells [120]. In addition, iron promotes the intrauterine growth of the fetus [121]. A deficiency, which in pregnant women is estimated for 50% of cases globally, reduces pro- and anti-inflammatory cytokines and may lead to anemia, morbidity, and prenatal mortality [122,123] and, in childhood, may contribute to the development of cognitive and behavioral problems [124,125]. In addition, low blood levels of Fe are related to altered functions of macrophages, NK cells, neutrophils, T and B cells [126]. Iron deficiency is associated with higher levels of prenatal depression [127,128]. Its integration can reduce the severity of respiratory viral infections and combat them [129].

**Selenium (Se).** Selenium is an essential micronutrient closely linked to the function of the immune system [124]. It shows immunostimulant effects on the proliferation of T and B cells and the activity of macrophages and NK cells [130,131]. Selenoproteins support the body’s defense system by influencing the functions of leukocytes [116]. Selenium deficiency is related to increased susceptibility to viral infections and, in pregnant women, may contribute to spontaneous abortion and preeclampsia [132]. The integration of this micronutrient increases resistance to viral infections, reduces the risk of prenatal mortality [133], and prevents hypertension [134]. Selenium intake also inhibits the development of postpartum depression, preventing its symptoms [135].

**Zinc (Zn).** Zinc plays a key role in modulating the immune system by promoting the development and function of macrophages, neutrophils, NK cells, inducing CD8+ T-cells proliferation, and assisting in the inflammatory process [120]. This micronutrient shows important antiviral properties, counteracting the replication of RNA viruses and inhibiting the replication of SARS-CoV at low concentrations [136]. Zinc deficiency is related to an altered phagocytic function, and reduced production of macrophages, DCs, and B and T cells [137]. Zn is essential for embryogenesis and fetal development. It is estimated that about 18% of pregnant women have a zinc deficiency. This can lead to premature birth, slowed fetal growth, increased risk of infection and also dwarfism [138], and alterations in brain development [139]. Integration of this micronutrient improves immune functions, protects against respiratory viral infections, reduces the risk of preterm birth, and lowers the incidence of gestational hypertension [140]. Zinc shows anxiolytic and antidepressant properties and its intake in higher quantities can better buffer the impact of stress on the development of symptoms of prenatal depression [141] and postpartum anxiety and depression [142].

**Vitamin A.** Vitamin A is a fat-soluble vitamin also called anti-inflammatory vitamin [116]. It plays an important role in the production, maturation, regulation, and function of macrophages, NK cells, neutrophils, innate lymphoid cells (ILCs), dendritic cells (DCs), T and B cells [143]. Its deficiency is related to respiratory infections [144], congenital fetal defects [145], gestational and diabetes mellitus [146], and schizophrenia [147]. Proper supplementation of this vitamin can reduce the risk of anemia [148] and shows to decrease the morbidity and mortality of some infectious diseases such as HIV [149]. Birth abnormalities have not been linked to vitamin A intakes of less than 10,000 IU per day during pregnancy. However, there are contradictory findings for daily doses ranging from 10,000 to 30,000 IU. Vitamin A intakes of more than 10,000 IU per day are not suggested for pregnant women who are well-nourished [150]. Moreover, excessive doses of vitamin A may be related to an increased risk of developing anxiety, depression, and mood disorders [151].

**Vitamin C.** Vitamin C, or ascorbic acid, is a water-soluble vitamin [152], which has high antioxidant activity and has the ability to fight infection. It modulates the migration of neutrophils to the site of infection and regulates phagocyte cells and NK cells [116]. In addition, it can mitigate the symptoms of respiratory infections [153]. Vitamin C is also able to promote intrauterine growth and childbirth [154]. Its integration contrasts preeclampsia, hypertension, and gestational diabetes [155]. With an average intake of 2000 IU/day of this vitamin, the risk of pregnancy-related complications, such as premature rupture of membranes, is decreased [156]. Vitamin C plays a key role in the biosynthesis of neurotransmitters such as serotonin and norepinephrine, lower levels of this vitamin have been found in patients with depression. Its intake also prevents schizophrenic and depressive symptoms [157,158].

**Figure 2:**
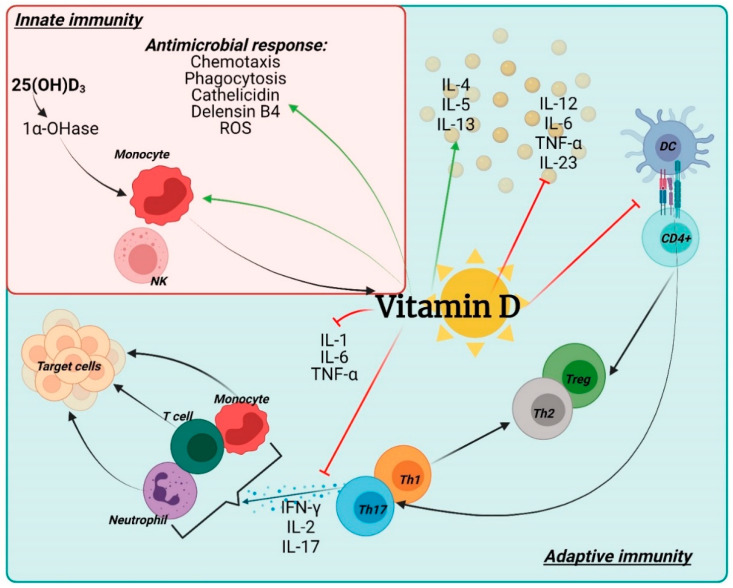
Vitamin D and its role on immune cells. The immunomodulatory effects of 1,25(OH)2D3 are depicted in this diagram. Different actors in the innate (red square) and adaptive immune (blue square) compartments are targeted by 1,25(OH)2D3. Vitamin D has been found to promote innate immune responses by increasing chemotaxis, antimicrobial peptides, and macrophage differentiation. Vitamin D also helps to enhance adaptive immune responses. For example, at the level of antigen-presenting cells, such as dendritic cells, vitamin D inhibits the surface expression of antigen complexed with MHCII, costimulatory molecules, and the production of cytokines IL12 and IL23, causing an indirect change in the Th1 and T-cell polarization. drives the Th17 phenotype towards a Th2 phenotype. Abbreviation: Th—T helper; NK—natural killer; DC—dendritic cells. Created with BioRender.com.

**Vitamin D.** Vitamin D is a fat-soluble vitamin capable of regulating calcium homeostasis in bones [116]; moreover, it has powerful anti-inflammatory and immunomodulatory activities on innate and adaptive immunity, stimulating the differentiation of monocytes-macrophages and inhibiting the production of cytokines [159,160] (Figure 2).

Calcium in extremely important for S100 protein expressed by immune cells, in particular macrophages, in immune response [161]. Vitamin D is related to a reduction in viral replication and viral infectivity [162,163]. Vitamin D in pregnant women has shown preventive effects against preeclampsia [164,165], and preterminal birth [166], playing a role in angiogenesis, placental implantation, and endothelial functions [167]. Vitamin D can regulate the immune system through its intracellular receptors (VDRs) present in monocytes, macrophages, B cells, T cells, DCs, and NKs [168]. It also modulates the synthesis of antimicrobial peptides, such as cathelicidin and defensins [169]. Vitamin D is also able to control and regulate the differentiation and function of NKs and DCs, promoting the presentation of antigens to lymphocytes [168]. Moreover, by reducing the secretion of IL-2, it leads to the suppression of Th lymphocytes, inhibiting the secretion of inflammatory cytokines such as IFNγ and IL-17 [168], helping to maintain a tolerogenic state [170,171]. Vitamin D modulates the proliferation of CD4+ lymphocytes [172] and promotes high levels of Treg cells, responsible for fetal tolerance [173]. Women with low vitamin D intake show a higher risk of developing anxiety and depressive symptoms early in pregnancy [174], with a higher probability of developing depression even after childbirth [175].

**Vitamin E.** Vitamin E is a fat-soluble vitamin with high antioxidant power and a high immunoregulatory capacity [176]. It modulates macrophages, NK cells, T cells, maturation and function of DCs, improves the humoral response, and reduces oxygen free radicals (ROS), nitrogen-free radicals (RNS), and prostaglandins [177,178]. Its integration improves the protection against viral infections and reduces the viral load of influenza [176,179]. In addition, this vitamin is capable to reduce and counteract oxidative stress during pregnancy, lowering the risk of preeclampsia and preterm birth [180]. Vitamin E levels in maternal blood are also correlated with improved fetal growth [181]. However, high doses of vitamin E (≥400 IU/day) are associated with an apparent decrease in birth weight, and its administration is suggested during the first trimester of pregnancy [182]. Low levels of vitamin E are also associated with an increased risk of depression, and its intake in the prenatal period could represent a protective factor against the development of depressive symptoms [183].

**Table 1:** Micronutrients helping pregnancy with recommended intake.

Micronutrients	Recommended Intake	Beneficial effects and Suggestions	References
Iron	16 mg/day	Strengthens immune functions, helps in resolving anemia and constricting viral infections. Better if administrated during the first and second trimesters.	[120,129]
Selenium	70–80 μg/day	Increases resistance to viral infections, reduces the risk of prenatal mortality, prevents hypertension, inhibits the development of postpartum depression.	[140,142]
Zinc	9.1 mg/day	Improves immune functions, protects against respiratory viral infections, reduces the risk of preterm birth, lowers the incidence of gestational hypertension, has antidepressant effect and may prevent teratogenesis.	[138,141]
Vitamin A	≤10,000 IU/day	Helpful for fetal development and for the regulation of the immune cells, but teratogenic at high doses.	[143,150]
Vitamin C	2000 IU/day	Mitigates the effects of respiratory syndromes, helps in the biosynthesis of serotonin and norepinephrine, protects from pregnancy-related complications.	[116,156]
Vitamin D	600–1500 IU/day	Implicated in the innate and adaptive immunity, protects against preeclampsia and preterminal birth.	[160,166]
Vitamin E	32.8–44.7 IU/day	Improves humoral response and protects against viral infections. Better if administrated at suggested doses during the first trimester of pregnancy.	[177,182]

## 5. Conclusions

Pregnancy involves changes in women’s immune system and psycho-physical status, which may make them more vulnerable to infection. During COVID-19, altered immune response and stress may contribute to facilitating SARS-CoV-2 infection, exacerbating the symptoms. The use of vitamins and micronutrients, usually taken during pregnancy, can help strengthen the immune system, mood, and psycho-physical state of pregnant women. In addition, they can counteract the severe symptoms of COVID-19, helping to prevent infection.

## Data Availability

Not applicable.

## References

[1] Aghaeepour N., Ganio E.A., McIlwain D., Tsai A.S., Tingle M., Van Gassen S., Gaudilliere D.K., Baca Q., McNeil L., Okada R. (2017). An immune clock of human pregnancy. Sci. Immunol..

[2] Mor G., Aldo P., Alvero A.B. (2017). The unique immunological and microbial aspects of pregnancy. Nat. Rev. Immunol..

[3] Marx G.F., Murthy P.K., Orkin L.R. (1970). Static compliance before and after vaginal delivery. Br. J. Anaesth..

[4] Chen M., Zeng J., Liu X., Sun G., Gao Y., Liao J., Yu J., Luo X., Qi H. (2020). Changes in physiology and immune system during pregnancy and coronavirus infection: A review. Eur. J. Obstet. Gynecol. Reprod. Biol..

[5] Hegewald M.J., Crapo R.O. (2011). Respiratory physiology in pregnancy. Clin. Chest Med..

[6] Jensen D., Wolfe L.A., Slatkovska L., Webb K.A., Davies G.A., O’Donnell D.E. (2005). Effects of human pregnancy on the ventilatory chemoreflex response to carbon dioxide. Am. J. Physiol. Regul. Integr. Comp. Physiol..

[7] Littauer E.Q., Skountzou I. (2018). Hormonal Regulation of Physiology, Innate Immunity and Antibody Response to H1N1 Influenza Virus Infection During Pregnancy. Front. Immunol..

[8] Kourtis A.P., Read J.S., Jamieson D.J. (2014). Pregnancy and infection. N. Engl. J. Med..

[9] Stevens G.A., Finucane M.M., De-Regil L.M., Paciorek C.J., Flaxman S.R., Branca F., Peña-Rosas J.P., Bhutta Z.A., Ezzati M. (2013). Global, regional, and national trends in haemoglobin concentration and prevalence of total and severe anaemia in children and pregnant and non-pregnant women for 1995–2011: A systematic analysis of population-representative data. Lancet Glob. Health.

[10] Muckenthaler M.U., Rivella S., Hentze M.W., Galy B. (2017). A Red Carpet for Iron Metabolism. Cell.

[11] Alesci A., Pergolizzi S., Lo Cascio P., Fumia A., Lauriano E.R. (2021). Neuronal regeneration: Vertebrates comparative overview and new perspectives for neurodegenerative diseases. Acta Zool..

[12] Fedullo A.L., Schiattarella A., Morlando M., Raguzzini A., Toti E., De Franciscis P., Peluso I. (2021). Mediterranean Diet for the Prevention of Gestational Diabetes in the COVID-19 Era: Implications of Il-6 In Diabesity. Int. J. Mol. Sci..

[13] Cascella M., Rajnik M., Aleem A., Dulebohn S.C., Di Napoli R. (2022). Features, Evaluation, and Treatment of Coronavirus (COVID-19).

[14] Chen N., Zhou M., Dong X., Qu J., Gong F., Han Y., Qiu Y., Wang J., Liu Y., Wei Y. (2020). Epidemiological and clinical characteristics of 99 cases of 2019 novel coronavirus pneumonia in Wuhan, China: A descriptive study. Lancet.

[15] Huang C., Wang Y., Li X., Ren L., Zhao J., Hu Y., Zhang L., Fan G., Xu J., Gu X. (2020). Clinical features of patients infected with 2019 novel coronavirus in Wuhan, China. Lancet.

[16] Hui D.S., Azhar E.I., Madani T.A., Ntoumi F., Kock R., Dar O., Ippolito G., McHugh T.D., Memish Z.A., Drosten C. (2020). The continuing 2019-nCoV epidemic threat of novel coronaviruses to global health—The latest 2019 novel coronavirus outbreak in Wuhan, China. Int. J. Infect. Dis..

[17] Buscarini E., Benedetti A., Monica F., Pasquale L., Buttitta F., Cameletti M., Ferrari C., Ricciardiello L. (2021). Changes in digestive cancer diagnosis during the SARS-CoV-2 pandemic in Italy: A nationwide survey. Dig. Liver. Dis..

[18] Wang Y., Shi L., Que J., Lu Q., Liu L., Lu Z., Xu Y., Liu J., Sun Y., Meng S. (2021). The impact of quarantine on mental health status among general population in China during the COVID-19 pandemic. Mol. Psychiatry.

[19] Zupo R., Castellana F., Sardone R., Sila A., Giagulli V.A., Triggiani V., Cincione R.I., Giannelli G., De Pergola G. (2020). Preliminary Trajectories in Dietary Behaviors during the COVID-19 Pandemic: A Public Health Call to Action to Face Obesity. Int. J. Environ. Res. Public Health.

[20] Khare J., Jindal S. (2020). Observational study on Effect of Lock Down due to COVID 19 on glycemic control in patients with Diabetes: Experience from Central India. Diabetes Metab. Syndr..

[21] Mattioli A.V., Sciomer S., Cocchi C., Maffei S., Gallina S. (2020). Quarantine during COVID-19 outbreak: Changes in diet and physical activity increase the risk of cardiovascular disease. Nutr. Metab. Cardiovasc. Dis..

[22] Martinez-Ferran M., de la Guia-Galipienso F., Sanchis-Gomar F., Pareja-Galeano H. (2020). Metabolic Impacts of Confinement during the COVID-19 Pandemic Due to Modified Diet and Physical Activity Habits. Nutrients.

[23] Schmidt C.B., Voorhorst I., van de Gaar V.H.W., Keukens A., Potter van Loon B.J., Snoek F.J., Honig A. (2019). Diabetes distress is associated with adverse pregnancy outcomes in women with gestational diabetes: A prospective cohort study. BMC Pregnancy Childbirth.

[24] Morais A.H.A., Aquino J.S., da Silva-Maia J.K., Vale S.H.L., Maciel B.L.L., Passos T.S. (2021). Nutritional status, diet and viral respiratory infections: Perspectives for severe acute respiratory syndrome coronavirus 2. Br. J. Nutr..

[25] Chaari A., Bendriss G., Zakaria D., McVeigh C. (2020). Importance of Dietary Changes During the Coronavirus Pandemic: How to Upgrade Your Immune Response. Front. Public Health.

[26] Richardson D.P., Lovegrove J.A. (2021). Nutritional status of micronutrients as a possible and modifiable risk factor for COVID-19: A UK perspective. Br. J. Nutr..

[27] Panyod S., Ho C.T., Sheen L.Y. (2020). Dietary therapy and herbal medicine for COVID-19 prevention: A review and perspective. J. Tradit. Complement. Med..

[28] Adams K.K., Baker W.L., Sobieraj D.M. (2020). Myth Busters: Dietary Supplements and COVID-19. Ann. Pharmacother..

[29] Konakci G., Ozgursoy Uran B.N., Erkin O. (2020). In the Turkish News: Coronavirus and "Alternative & complementary" medicine methods. Complement. Ther. Med..

[30] Alesci A., Fumia A., Lo Cascio P., Miller A., Cicero N. (2021). Immunostimulant and Antidepressant Effect of Natural Compounds in the Management of COVID-19 Symptoms. J. Am. Coll. Nutr..

[31] Alesci A., Aragona M., Cicero N., Lauriano E.R. (2022). Can nutraceuticals assist treatment and improve COVID-19 symptoms?. Nat. Prod. Res..

[32] Marino A., Santoro G., Spataro F., Lauriano E.R., Pergolizzi S., Cimino F., Speciale A., Nostro A., Bisignano G., Dugo G. (2013). Resveratrol role in Staphylococcus aureus-induced corneal inflammation. Pathog. Dis..

[33] Alesci A., Cicero N., Salvo A., Palombieri D., Zaccone D., Dugo G., Bruno M., Vadala R., Lauriano E.R., Pergolizzi S. (2014). Extracts deriving from olive mill waste water and their effects on the liver of the goldfish Carassius auratus fed with hypercholesterolemic diet. Nat. Prod. Res..

[34] Alesci A., Salvo A., Lauriano E.R., Gervasi T., Palombieri D., Bruno M., Pergolizzi S., Cicero N. (2015). Production and extraction of astaxanthin from Phaffia rhodozyma and its biological effect on alcohol-induced renal hypoxia in Carassius auratus. Nat. Prod. Res..

[35] Alessio A., Pergolizzi S., Gervasi T., Aragona M., Lo Cascio P., Cicero N., Lauriano E.R. (2020). Biological effect of astaxanthin on alcohol-induced gut damage in Carassius auratus used as experimental model. Nat. Prod. Res..

[36] Alesci A., Nicosia N., Fumia A., Giorgianni F., Santini A., Cicero N. (2022). Resveratrol and Immune Cells: A Link to Improve Human Health. Molecules.

[37] Aghraz A., Gonçalves S., Rodríguez-Solana R., Dra L.A., Di Stefano V., Dugo G., Cicero N., Larhsini M., Markouk M., Romano A. (2018). Antioxidant activity and enzymes inhibitory properties of several extracts from two Moroccan Asteraceae species. South Afr. J. Bot..

[38] Fumia A., Cicero N., Gitto M., Nicosia N., Alesci A. (2021). Role of nutraceuticals on neurodegenerative diseases: Neuroprotective and immunomodulant activity. Nat. Prod. Res..

[39] Alesci A., Miller A., Tardugno R., Pergolizzi S. (2022). Chemical analysis, biological and therapeutic activities of *Olea europaea* L. extracts. Nat. Prod. Res..

[40] Alesci A., Lauriano E.R., Fumia A., Irrera N., Mastrantonio E., Vaccaro M., Gangemi S., Santini A., Cicero N., Pergolizzi S. (2022). Relationship between Immune Cells, Depression, Stress, and Psoriasis: Could the Use of Natural Products Be Helpful?. Molecules.

[41] Allegra A., Tonacci A., Pioggia G., Musolino C., Gangemi S. (2020). Vitamin deficiency as risk factor for SARS-CoV-2 infection: Correlation with susceptibility and prognosis. Eur. Rev. Med. Pharmacol. Sci..

[42] Ellington S., Strid P., Tong V.T., Woodworth K., Galang R.R., Zambrano L.D., Nahabedian J., Anderson K., Gilboa S.M. (2020). Characteristics of Women of Reproductive Age with Laboratory-Confirmed SARS-CoV-2 Infection by Pregnancy Status—United States, January 22-June 7, 2020. MMWR Morb. Mortal. Wkly. Rep..

[43] Adhikari E.H., Moreno W., Zofkie A.C., MacDonald L., McIntire D.D., Collins R.R.J., Spong C.Y. (2020). Pregnancy Outcomes Among Women With and Without Severe Acute Respiratory Syndrome Coronavirus 2 Infection. JAMA Netw. Open.

[44] Vivanti A.J., Vauloup-Fellous C., Prevot S., Zupan V., Suffee C., Do Cao J., Benachi A., De Luca D. (2020). Transplacental transmission of SARS-CoV-2 infection. Nat. Commun..

[45] Hosier H., Farhadian S.F., Morotti R.A., Deshmukh U., Lu-Culligan A., Campbell K.H., Yasumoto Y., Vogels C.B., Casanovas-Massana A., Vijayakumar P. (2020). SARS-CoV-2 infection of the placenta. J. Clin. Invest..

[46] Prochaska E., Jang M., Burd I. (2020). COVID-19 in pregnancy: Placental and neonatal involvement. Am. J. Reprod. Immunol..

[47] Algarroba G.N., Rekawek P., Vahanian S.A., Khullar P., Palaia T., Peltier M.R., Chavez M.R., Vintzileos A.M. (2020). Visualization of severe acute respiratory syndrome coronavirus 2 invading the human placenta using electron microscopy. Am. J. Obstet. Gynecol..

[48] Zeng H., Xu C., Fan J., Tang Y., Deng Q., Zhang W., Long X. (2020). Antibodies in Infants Born to Mothers With COVID-19 Pneumonia. JAMA.

[49] Dong L., Tian J., He S., Zhu C., Wang J., Liu C., Yang J. (2020). Possible Vertical Transmission of SARS-CoV-2 From an Infected Mother to Her Newborn. JAMA.

[50] Gajbhiye R.K., Modi D.N., Mahale S.D. (2020). Pregnancy outcomes, Newborn complications and Maternal-Fetal Transmission of SARS-CoV-2 in women with COVID-19: A systematic review. Preprint.

[51] Li M., Chen L., Zhang J., Xiong C., Li X. (2020). The SARS-CoV-2 receptor ACE2 expression of maternal-fetal interface and fetal organs by single-cell transcriptome study. PLoS ONE.

[52] Alzamora M.C., Paredes T., Caceres D., Webb C.M., Valdez L.M., La Rosa M. (2020). Severe COVID-19 during Pregnancy and Possible Vertical Transmission. Am. J. Perinatol..

[53] Figueiredo A.S., Schumacher A. (2016). The T helper type 17/regulatory T cell paradigm in pregnancy. Immunology.

[54] Jorgensen N., Persson G., Hviid T.V.F. (2019). The Tolerogenic Function of Regulatory T Cells in Pregnancy and Cancer. Front. Immunol..

[55] Muyayalo K.P., Li Z.H., Mor G., Liao A.H. (2018). Modulatory effect of intravenous immunoglobulin on Th17/Treg cell balance in women with unexplained recurrent spontaneous abortion. Am. J. Reprod. Immunol..

[56] Chen Y., Li Z., Zhang Y.Y., Zhao W.H., Yu Z.Y. (2020). Maternal health care management during the outbreak of coronavirus disease 2019. J. Med. Virol..

[57] Marzi M., Vigano A., Trabattoni D., Villa M.L., Salvaggio A., Clerici E., Clerici M. (1996). Characterization of type 1 and type 2 cytokine production profile in physiologic and pathologic human pregnancy. Clin. Exp. Immunol..

[58] Le Gars M., Kay A.W., Bayless N.L., Aziz N., Dekker C.L., Swan G.E., Davis M.M., Blish C.A. (2016). Increased Proinflammatory Responses of Monocytes and Plasmacytoid Dendritic Cells to Influenza A Virus Infection during Pregnancy. J. Infect. Dis..

[59] Mullins E., Evans D., Viner R.M., O’Brien P., Morris E. (2020). Coronavirus in pregnancy and delivery: Rapid review. Ultrasound Obstet. Gynecol..

[60] Jamieson D.J., Honein M.A., Rasmussen S.A., Williams J.L., Swerdlow D.L., Biggerstaff M.S., Lindstrom S., Louie J.K., Christ C.M., Bohm S.R. (2009). H1N1 2009 influenza virus infection during pregnancy in the USA. Lancet.

[61] Zaigham M., Andersson O. (2020). Maternal and perinatal outcomes with COVID-19: A systematic review of 108 pregnancies. Acta Obstet. Gynecol. Scand..

[62] Ryan G.A., Purandare N.C., McAuliffe F.M., Hod M., Purandare C.N. (2020). Clinical update on COVID-19 in pregnancy: A review article. J. Obstet. Gynecol. Res..

[63] Chen G., Liao Q., Ai J., Yang B., Bai H., Chen J., Liu F., Cao Y., Liu H., Li K. (2021). Immune Response to COVID-19 During Pregnancy. Front. Immunol..

[64] Phiel K.L., Henderson R.A., Adelman S.J., Elloso M.M. (2005). Differential estrogen receptor gene expression in human peripheral blood mononuclear cell populations. Immunol. Lett..

[65] Mauvais-Jarvis F., Klein S.L., Levin E.R. (2020). Estradiol, Progesterone, Immunomodulation, and COVID-19 Outcomes. Endocrinology.

[66] Straub R.H. (2007). The complex role of estrogens in inflammation. Endocr. Rev..

[67] Channappanavar R., Fett C., Mack M., Ten Eyck P.P., Meyerholz D.K., Perlman S. (2017). Sex-Based Differences in Susceptibility to Severe Acute Respiratory Syndrome Coronavirus Infection. J. Immunol..

[68] Shah N.M., Lai P.F., Imami N., Johnson M.R. (2019). Progesterone-Related Immune Modulation of Pregnancy and Labor. Front. Endocrinol..

[69] Hall O.J., Limjunyawong N., Vermillion M.S., Robinson D.P., Wohlgemuth N., Pekosz A., Mitzner W., Klein S.L. (2016). Progesterone-Based Therapy Protects Against Influenza by Promoting Lung Repair and Recovery in Females. PLoS Pathog..

[70] Gordon D.E., Jang G.M., Bouhaddou M., Xu J., Obernier K., White K.M., O’Meara M.J., Rezelj V.V., Guo J.Z., Swaney D.L. (2020). A SARS-CoV-2 protein interaction map reveals targets for drug repurposing. Nature.

[71] Elshafeey F., Magdi R., Hindi N., Elshebiny M., Farrag N., Mahdy S., Sabbour M., Gebril S., Nasser M., Kamel M. (2020). A systematic scoping review of COVID-19 during pregnancy and childbirth. Int. J. Gynaecol. Obstet..

[72] Liu H., Wang L.L., Zhao S.J., Kwak-Kim J., Mor G., Liao A.H. (2020). Why are pregnant women susceptible to COVID-19? An immunological viewpoint. J. Reprod. Immunol..

[73] Doria A., Iaccarino L., Arienti S., Ghirardello A., Zampieri S., Rampudda M.E., Cutolo M., Tincani A., Todesco S. (2006). Th2 immune deviation induced by pregnancy: The two faces of autoimmune rheumatic diseases. Reprod. Toxicol..

[74] Qiancheng X., Jian S., Lingling P., Lei H., Xiaogan J., Weihua L., Gang Y., Shirong L., Zhen W., GuoPing X. (2020). Coronavirus disease 2019 in pregnancy. Int. J. Infect. Dis..

[75] Hantoushzadeh S., Shamshirsaz A.A., Aleyasin A., Seferovic M.D., Aski S.K., Arian S.E., Pooransari P., Ghotbizadeh F., Aalipour S., Soleimani Z. (2020). Maternal death due to COVID-19. Am. J. Obstet. Gynecol..

[76] Wu C., Yang W., Wu X., Zhang T., Zhao Y., Ren W., Xia J. (2020). Clinical Manifestation and Laboratory Characteristics of SARS-CoV-2 Infection in Pregnant Women. Virol. Sin..

[77] Alesci A., Lauriano E.R., Aragona M., Capillo G., Pergolizzi S. (2020). Marking vertebrates langerhans cells, from fish to mammals. Acta Histochem..

[78] Pergolizzi S., Rizzo G., Favaloro A., Alesci A., Pallio S., Melita G., Cutroneo G., Lauriano E.R. (2021). Expression of VAChT and 5-HT in Ulcerative colitis dendritic cells. Acta Histochem..

[79] Pallio G., Bitto A., Ieni A., Irrera N., Mannino F., Pallio S., Altavilla D., Squadrito F., Scarpignato C., Minutoli L. (2020). Combined Treatment with Polynucleotides and Hyaluronic Acid Improves Tissue Repair in Experimental Colitis. Biomedicines.

[80] Kraus T.A., Engel S.M., Sperling R.S., Kellerman L., Lo Y., Wallenstein S., Escribese M.M., Garrido J.L., Singh T., Loubeau M. (2012). Characterizing the pregnancy immune phenotype: Results of the viral immunity and pregnancy (VIP) study. J. Clin. Immunol..

[81] Lauriano E.R., Pergolizzi S., Capillo G., Kuciel M., Alesci A., Faggio C. (2016). Immunohistochemical characterization of Toll-like receptor 2 in gut epithelial cells and macrophages of goldfish Carassius auratus fed with a high-cholesterol diet. Fish Shellfish Immunol..

[82] Lauriano E.R., Silvestri G., Kuciel M., Zuwala K., Zaccone D., Palombieri D., Alesci A., Pergolizzi S. (2014). Immunohistochemical localization of Toll-like receptor 2 in skin Langerhans’ cells of striped dolphin (*Stenella coeruleoalba*). Tissue Cell.

[83] Lauriano E.R., Aragona M., Alesci A., Lo Cascio P., Pergolizzi S. (2021). Toll-Like Receptor 2 and α-Smooth Muscle Actin expressed in the tunica of a urochordate, Styela plicata. Tissue Cell.

[84] Alesci A., Pergolizzi S., Capillo G., Lo Cascio P., Lauriano E.R. (2022). Rodlet cells in kidney of goldfish (*Carassius auratus*, Linnaeus 1758): A light and confocal microscopy study. Acta Histochem..

[85] Alesci A., Pergolizzi S., Fumia A., Calabrò C., Lo Cascio P., Lauriano E.R. (2022). Mast cells in goldfish (*Carassius auratus*) gut: Immunohistochemical characterization. Acta Zool..

[86] Alesci A., Pergolizzi S., Lo Cascio P., Capillo G., Lauriano E.R. (2022). Localization of vasoactive intestinal peptide and toll-like receptor 2 immunoreactive cells in endostyle of urochordate Styela plicata (Lesueur, 1823). Microsc. Res. Tech..

[87] Marino A., Pergolizzi S., Lauriano E.R., Santoro G., Spataro F., Cimino F., Speciale A., Nostro A., Bisignano G. (2015). TLR2 activation in corneal stromal cells by Staphylococcus aureus-induced keratitis. APMIS.

[88] Areia A.L., Mota-Pinto A. (2020). Can immunity during pregnancy influence SARS-CoV-2 infection?—A systematic review. J. Reprod. Immunol..

[89] Di Mascio D., Khalil A., Saccone G., Rizzo G., Buca D., Liberati M., Vecchiet J., Nappi L., Scambia G., Berghella V. (2020). Outcome of coronavirus spectrum infections (SARS, MERS, COVID-19) during pregnancy: A systematic review and meta-analysis. Am. J. Obstet. Gynecol. MFM.

[90] Chen H., Guo J., Wang C., Luo F., Yu X., Zhang W., Li J., Zhao D., Xu D., Gong Q. (2020). Clinical characteristics and intrauterine vertical transmission potential of COVID-19 infection in nine pregnant women: A retrospective review of medical records. Lancet.

[91] Zhao S., Xie T., Shen L., Liu H., Wang L., Ma X., Wu J., Yuan S., Mor G., Liao A. (2021). An Immunological Perspective: What Happened to Pregnant Women After Recovering From COVID-19?. Front. Immunol..

[92] Chen Z., John Wherry E. (2020). T cell responses in patients with COVID-19. Nat. Rev. Immunol..

[93] Ragab D., Salah Eldin H., Taeimah M., Khattab R., Salem R. (2020). The COVID-19 Cytokine Storm; What We Know So Far. Front. Immunol..

[94] Grifoni A., Weiskopf D., Ramirez S.I., Mateus J., Dan J.M., Moderbacher C.R., Rawlings S.A., Sutherland A., Premkumar L., Jadi R.S. (2020). Targets of T Cell Responses to SARS-CoV-2 Coronavirus in Humans with COVID-19 Disease and Unexposed Individuals. Cell.

[95] Wen W., Su W., Tang H., Le W., Zhang X., Zheng Y., Liu X., Xie L., Li J., Ye J. (2020). Immune cell profiling of COVID-19 patients in the recovery stage by single-cell sequencing. Cell Discov..

[96] Alhusen J.L., Ayres L., DePriest K. (2016). Effects of Maternal Mental Health on Engagement in Favorable Health Practices During Pregnancy. J. Midwifery Womens Health.

[97] Cetin O., Guzel Ozdemir P., Kurdoglu Z., Sahin H.G. (2017). Investigation of maternal psychopathological symptoms, dream anxiety and insomnia in preeclampsia. J. Matern. Fetal Neonatal Med..

[98] Woody C.A., Ferrari A.J., Siskind D.J., Whiteford H.A., Harris M.G. (2017). A systematic review and meta-regression of the prevalence and incidence of perinatal depression. J. Affect. Disord..

[99] Gariepy A.M., Lundsberg L.S., Miller D., Stanwood N.L., Yonkers K.A. (2016). Are pregnancy planning and pregnancy timing associated with maternal psychiatric illness, psychological distress and support during pregnancy?. J. Affect. Disord..

[100] Caparros-Gonzalez R.A., Romero-Gonzalez B., Strivens-Vilchez H., Gonzalez-Perez R., Martinez-Augustin O., Peralta-Ramirez M.I. (2017). Hair cortisol levels, psychological stress and psychopathological symptoms as predictors of postpartum depression. PLoS ONE.

[101] Romero-Gonzalez B., Caparros-Gonzalez R.A., Gonzalez-Perez R., Delgado-Puertas P., Peralta-Ramirez M.I. (2018). Newborn infants’ hair cortisol levels reflect chronic maternal stress during pregnancy. PLoS ONE.

[102] Ma Z.F., Zhang Y., Luo X., Li X., Li Y., Liu S., Zhang Y. (2020). Increased stressful impact among general population in mainland China amid the COVID-19 pandemic: A nationwide cross-sectional study conducted after Wuhan city’s travel ban was lifted. Int. J. Soc. Psychiatry.

[103] Saccone G., Florio A., Aiello F., Venturella R., De Angelis M.C., Locci M., Bifulco G., Zullo F., Di Spiezio Sardo A. (2020). Psychological impact of coronavirus disease 2019 in pregnant women. Am. J. Obstet. Gynecol..

[104] Davenport M.H., Meyer S., Meah V.L., Strynadka M.C., Khurana R. (2020). Moms Are Not OK: COVID-19 and Maternal Mental Health. Front. Glob. Women’s Health.

[105] Zhang Y., Ma Z.F. (2021). Psychological responses and lifestyle changes among pregnant women with respect to the early stages of COVID-19 pandemic. Int. J. Soc. Psychiatry.

[106] Caffieri A., Margherita G. (2021). The psychological impact of COVID-19 on women’s wellbeing during pregnancy and postpartum one year after pandemic outbreak in Italy. A Systematic review. Mediterr. J. Clin. Psychol..

[107] Schimmenti A., Billieux J., Starcevic V. (2020). The four horsemen of fear: An integrated model of understanding fear experiences during the COVID-19 pandemic. Clin. Neuropsychiatry.

[108] Ravaldi C., Wilson A., Ricca V., Homer C., Vannacci A. (2021). Pregnant women voice their concerns and birth expectations during the COVID-19 pandemic in Italy. Women Birth.

[109] Cena L., Biban P., Janos J., Lavelli M., Langfus J., Tsai A., Youngstrom E.A., Stefana A. (2021). The Collateral Impact of COVID-19 Emergency on Neonatal Intensive Care Units and Family-Centered Care: Challenges and Opportunities. Front. Psychol..

[110] O’Hara M.W., Wisner K.L. (2014). Perinatal mental illness: Definition, description and aetiology. Best Pract. Res. Clin. Obstet. Gynaecol..

[111] Liu C.H., Erdei C., Mittal L. (2021). Risk factors for depression, anxiety, and PTSD symptoms in perinatal women during the COVID-19 Pandemic. Psychiatry Res..

[112] Khoury J.E., Atkinson L., Bennett T., Jack S.M., Gonzalez A. (2021). COVID-19 and mental health during pregnancy: The importance of cognitive appraisal and social support. J. Affect. Disord..

[113] Heller H.M., Hoogendoorn A.W., Honig A., Broekman B.F.P., van Straten A. (2020). The Effectiveness of a Guided Internet-Based Tool for the Treatment of Depression and Anxiety in Pregnancy (MamaKits Online): Randomized Controlled Trial. J. Med. Internet Res..

[114] Sani G., Janiri D., Di Nicola M., Janiri L., Ferretti S., Chieffo D. (2020). Mental health during and after the COVID-19 emergency in Italy. Psychiatry Clin. Neurosci..

[115] Yazdanimehr R., Omidi A., Sadat Z., Akbari H. (2016). The Effect of Mindfulness-integrated Cognitive Behavior Therapy on Depression and Anxiety among Pregnant Women: A Randomized Clinical Trial. J. Caring Sci..

[116] Nawsherwan, Khan S., Zeb F., Shoaib M., Nabi G., Ul Haq I., Xu K., Li H. (2020). Selected Micronutrients: An Option to Boost Immunity against COVID-19 and Prevent Adverse Pregnancy Outcomes in Pregnant Women: A Narrative Review. Iran, J. Public Health.

[117] Gernand A.D., Schulze K.J., Stewart C.P., West K.P., Christian P. (2016). Micronutrient deficiencies in pregnancy worldwide: Health effects and prevention. Nat. Rev. Endocrinol..

[118] Alpert P.T. (2017). The Role of Vitamins and Minerals on the Immune System. Home Health Care Manag. Pract..

[119] Wilson R.L., Gummow J.A., McAninch D., Bianco-Miotto T., Roberts C.T. (2018). Vitamin and mineral supplementation in pregnancy: Evidence to practice. J. Pharm. Pract. Res..

[120] Maggini S., Pierre A., Calder P.C. (2018). Immune Function and Micronutrient Requirements Change over the Life Course. Nutrients.

[121] Osendarp S.J., Murray-Kolb L.E., Black M.M. (2010). Case study on iron in mental development—In memory of John Beard (1947–2009). Nutr. Rev..

[122] Guo Y., Zhang N., Zhang D., Ren Q., Ganz T., Liu S., Nemeth E. (2019). Iron homeostasis in pregnancy and spontaneous abortion. Am. J. Hematol..

[123] Chikakuda A.T., Shin D., Comstock S.S., Song S., Song W.O. (2018). Compliance to Prenatal Iron and Folic Acid Supplement Use in Relation to Low Birth Weight in Lilongwe, Malawi. Nutrients.

[124] Farias P.M., Marcelino G., Santana L.F., de Almeida E.B., Guimaraes R.C.A., Pott A., Hiane P.A., Freitas K.C. (2020). Minerals in Pregnancy and Their Impact on Child Growth and Development. Molecules.

[125] McKeating D.R., Fisher J.J., Perkins A.V. (2019). Elemental Metabolomics and Pregnancy Outcomes. Nutrients.

[126] Kuvibidila S., Porretta C., Baliga S. (2014). Aneuploidy assessed by DNA index influences the effect of iron status on plasma and/or supernatant cytokine levels and progression of cells through the cell cycle in a mouse model. Cytokine.

[127] Dama M., Van Lieshout R.J., Mattina G., Steiner M. (2018). Iron Deficiency and Risk of Maternal Depression in Pregnancy: An Observational Study. J. Obstet. Gynaecol. Can..

[128] Bae H.S., Kim S.Y., Ahnv H.S., Cho Y.K. (2010). Comparison of nutrient intake, life style variables, and pregnancy outcomes by the depression degree of pregnant women. Nutr. Res. Pract..

[129] Oppenheimer S.J. (2001). Iron and its relation to immunity and infectious disease. J. Nutr..

[130] Huang Z., Rose A.H., Hoffmann P.R. (2012). The role of selenium in inflammation and immunity: From molecular mechanisms to therapeutic opportunities. Antioxid. Redox. Signal.

[131] Avery J.C., Hoffmann P.R. (2018). Selenium, Selenoproteins, and Immunity. Nutrients.

[132] Jiang S., Yang B., Xu J., Liu Z., Yan C., Zhang J., Li S., Shen X. (2019). Associations of Internal-Migration Status with Maternal Exposure to Stress, Lead, and Selenium Deficiency Among Pregnant Women in Shanghai, China. Biol. Trace Elem. Res..

[133] Fialova L., Malbohan I., Kalousova M., Soukupova J., Krofta L., Stipek S., Zima T. (2006). Oxidative stress and inflammation in pregnancy. Scand. J. Clin. Lab. Invest..

[134] Nawrot T.S., Staessen J.A., Roels H.A., Den Hond E., Thijs L., Fagard R.H., Dominiczak A.F., Struijker-Boudier H.A. (2007). Blood pressure and blood selenium: A cross-sectional and longitudinal population study. Eur. Heart J..

[135] Mokhber N., Namjoo M., Tara F., Boskabadi H., Rayman M.P., Ghayour-Mobarhan M., Sahebkar A., Majdi M.R., Tavallaie S., Azimi-Nezhad M. (2011). Effect of supplementation with selenium on postpartum depression: A randomized double-blind placebo-controlled trial. J. Matern. Fetal Neonatal Med..

[136] te Velthuis A.J., van den Worm S.H., Sims A.C., Baric R.S., Snijder E.J., van Hemert M.J. (2010). Zn(2+) inhibits coronavirus and arterivirus RNA polymerase activity in vitro and zinc ionophores block the replication of these viruses in cell culture. PLoS Pathog..

[137] Bonaventura P., Benedetti G., Albarede F., Miossec P. (2015). Zinc and its role in immunity and inflammation. Autoimmun. Rev..

[138] Stammers A.L., Lowe N.M., Medina M.W., Patel S., Dykes F., Perez-Rodrigo C., Serra-Majam L., Nissensohn M., Moran V.H. (2015). The relationship between zinc intake and growth in children aged 1-8 years: A systematic review and meta-analysis. Eur. J. Clin. Nutr..

[139] Sweetman D.U., O’Donnell S.M., Lalor A., Grant T., Greaney H. (2019). Zinc and vitamin A deficiency in a cohort of children with autism spectrum disorder. Child Care Health Dev..

[140] King J.C. (2011). Zinc: An essential but elusive nutrient. Am. J. Clin. Nutr..

[141] Roy A., Evers S.E., Avison W.R., Campbell M.K. (2010). Higher zinc intake buffers the impact of stress on depressive symptoms in pregnancy. Nutr. Res..

[142] Fard F.E., Mirghafourvand M., Mohammad-Alizadeh Charandabi S., Farshbaf-Khalili A., Javadzadeh Y., Asgharian H. (2017). Effects of zinc and magnesium supplements on postpartum depression and anxiety: A randomized controlled clinical trial. Women Health.

[143] McCullough F.S., Northrop-Clewes C.A., Thurnham D.I. (1999). The effect of vitamin A on epithelial integrity. Proc. Nutr. Soc..

[144] Huang Z., Liu Y., Qi G., Brand D., Zheng S.G. (2018). Role of Vitamin A in the Immune System. J. Clin. Med..

[145] Hammouda S.A., Abd Al-Halim O.A., Mohamadin A.M. (2013). Serum levels of some micronutrients and congenital malformations: A prospective cohort study in healthy saudi-arabian first-trimester pregnant women. Int. J. Vitam. Nutr. Res..

[146] Lira L.Q., Dimenstein R. (2010). Vitamin A and gestational diabetes. Rev. Assoc. Med. Bras..

[147] Bao Y., Ibram G., Blaner W.S., Quesenberry C.P., Shen L., McKeague I.W., Schaefer C.A., Susser E.S., Brown A.S. (2012). Low maternal retinol as a risk factor for schizophrenia in adult offspring. Schizophr. Res..

[148] Bastos Maia S., Rolland Souza A.S., Costa Caminha M.F., Lins da Silva S., Callou Cruz R., Carvalho Dos Santos C., Batista Filho M. (2019). Vitamin A and Pregnancy: A Narrative Review. Nutrients.

[149] Mora J.R., Iwata M., von Andrian U.H. (2008). Vitamin effects on the immune system: Vitamins A and D take centre stage. Nat. Rev. Immunol..

[150] Dibley M.J., Jeacocke D.A. (2016). Safety and Toxicity of Vitamin A Supplements in Pregnancy. Food Nutr. Bull..

[151] Chapman M.S. (2012). Vitamin a: History, current uses, and controversies. Semin. Cutan. Med. Surg..

[152] Harrison F.E., Bowman G.L., Polidori M.C. (2014). Ascorbic acid and the brain: Rationale for the use against cognitive decline. Nutrients.

[153] Hemila H. (2003). Vitamin C and SARS coronavirus. J. Antimicrob. Chemother..

[154] Biondi C., Pavan B., Dalpiaz A., Medici S., Lunghi L., Vesce F. (2007). Expression and characterization of vitamin C transporter in the human trophoblast cell line HTR-8/SVneo: Effect of steroids, flavonoids and NSAIDs. Mol. Hum. Reprod..

[155] Chappell L.C., Seed P.T., Kelly F.J., Briley A., Hunt B.J., Charnock-Jones D.S., Mallet A., Poston L. (2002). Vitamin C and E supplementation in women at risk of preeclampsia is associated with changes in indices of oxidative stress and placental function. Am. J. Obstet. Gynecol..

[156] Rumbold A., Ota E., Nagata C., Shahrook S., Crowther C.A. (2015). Vitamin C supplementation in pregnancy. Cochrane Database Syst. Rev..

[157] Payne M.E., Steck S.E., George R.R., Steffens D.C. (2012). Fruit, vegetable, and antioxidant intakes are lower in older adults with depression. J. Acad. Nutr. Diet..

[158] Ramsey D., Muskin P.R. (2013). Vitamin deficiencies and mental health: How are they linked. Curr. Pschiatry.

[159] Chun R.F., Liu P.T., Modlin R.L., Adams J.S., Hewison M. (2014). Impact of vitamin D on immune function: Lessons learned from genome-wide analysis. Front. Physiol..

[160] Siddiqui M., Manansala J.S., Abdulrahman H.A., Nasrallah G.K., Smatti M.K., Younes N., Althani A.A., Yassine H.M. (2020). Immune Modulatory Effects of Vitamin D on Viral Infections. Nutrients.

[161] Alesci A., Cicero N., Fumia A., Petrarca C., Mangifesta R., Nava V., Lo Cascio P., Gangemi S., Di Gioacchino M., Lauriano E.R. (2022). Histological and Chemical Analysis of Heavy Metals in Kidney and Gills of Boops boops: Melanomacrophages Centers and Rodlet Cells as Environmental Biomarkers. Toxics.

[162] Bezerra Espinola M.S., Bertelli M., Bizzarri M., Unfer V., Lagana A.S., Visconti B., Aragona C. (2021). Inositol and vitamin D may naturally protect human reproduction and women undergoing assisted reproduction from COVID-19 risk. J. Reprod. Immunol..

[163] Teymoori-Rad M., Shokri F., Salimi V., Marashi S.M. (2019). The interplay between vitamin D and viral infections. Rev. Med. Virol..

[164] Bodnar L.M., Catov J.M., Simhan H.N., Holick M.F., Powers R.W., Roberts J.M. (2007). Maternal vitamin D deficiency increases the risk of preeclampsia. J. Clin. Endocrinol. Metab..

[165] Baker A.M., Haeri S., Camargo C.A., Espinola J.A., Stuebe A.M. (2010). A nested case-control study of midgestation vitamin D deficiency and risk of severe preeclampsia. J. Clin. Endocrinol. Metab..

[166] Perez-Ferre N., Torrejon M.J., Fuentes M., Fernandez M.D., Ramos A., Bordiu E., del Valle L., Rubio M.A., Bedia A.R., Montanez C. (2012). Association of low serum 25-hydroxyvitamin D levels in pregnancy with glucose homeostasis and obstetric and newborn outcomes. Endocr. Pract..

[167] Wei S.Q. (2014). Vitamin D and pregnancy outcomes. Curr. Opin. Obstet. Gynecol..

[168] Cyprian F., Lefkou E., Varoudi K., Girardi G. (2019). Immunomodulatory Effects of Vitamin D in Pregnancy and Beyond. Front. Immunol..

[169] Wang T.T., Nestel F.P., Bourdeau V., Nagai Y., Wang Q., Liao J., Tavera-Mendoza L., Lin R., Hanrahan J.W., Mader S. (2004). Cutting edge: 1,25-dihydroxyvitamin D3 is a direct inducer of antimicrobial peptide gene expression. J. Immunol..

[170] Bartels L.E., Hvas C.L., Agnholt J., Dahlerup J.F., Agger R. (2010). Human dendritic cell antigen presentation and chemotaxis are inhibited by intrinsic 25-hydroxy vitamin D activation. Int. Immunopharmacol..

[171] Murdaca G., Tonacci A., Negrini S., Greco M., Borro M., Puppo F., Gangemi S. (2019). Emerging role of vitamin D in autoimmune diseases: An update on evidence and therapeutic implications. Autoimmun. Rev..

[172] Palmer M.T., Lee Y.K., Maynard C.L., Oliver J.R., Bikle D.D., Jetten A.M., Weaver C.T. (2011). Lineage-specific effects of 1,25-dihydroxyvitamin D(3) on the development of effector CD4 T cells. J. Biol. Chem..

[173] Chambers E.S., Hawrylowicz C.M. (2011). The impact of vitamin D on regulatory T cells. Curr. Allergy Asthma Rep..

[174] Huang J.Y., Arnold D., Qiu C.F., Miller R.S., Williams M.A., Enquobahrie D.A. (2014). Association of serum vitamin D with symptoms of depression and anxiety in early pregnancy. J. Womens Health (Larchmt).

[175] Gur E.B., Gokduman A., Turan G.A., Tatar S., Hepyilmaz I., Zengin E.B., Eskicioglu F., Guclu S. (2014). Mid-pregnancy vitamin D levels and postpartum depression. Eur. J. Obstet. Gynecol. Reprod. Biol..

[176] Lee G.Y., Han S.N. (2018). The Role of Vitamin E in Immunity. Nutrients.

[177] Mosser D.M., Edwards J.P. (2008). Exploring the full spectrum of macrophage activation. Nat. Rev. Immunol..

[178] Tan P.H., Sagoo P., Chan C., Yates J.B., Campbell J., Beutelspacher S.C., Foxwell B.M., Lombardi G., George A.J. (2005). Inhibition of NF-kappa B and oxidative pathways in human dendritic cells by antioxidative vitamins generates regulatory T cells. J. Immunol..

[179] Han S.N., Wu D., Ha W.K., Beharka A., Smith D.E., Bender B.S., Meydani S.N. (2000). Vitamin E supplementation increases T helper 1 cytokine production in old mice infected with influenza virus. Immunology.

[180] Scholl T.O., Leskiw M., Chen X., Sims M., Stein T.P. (2005). Oxidative stress, diet, and the etiology of preeclampsia. Am. J. Clin. Nutr..

[181] Cave C., Hanson C., Schumacher M., Lyden E., Furtado J., Obaro S., Delair S., Kocmich N., Rezac A., Izevbigie N.I. (2018). A Comparison of Vitamin E Status and Associated Pregnancy Outcomes in Maternal(-)Infant Dyads between a Nigerian and a United States Population. Nutrients.

[182] Boskovic R., Gargaun L., Oren D., Djulus J., Koren G. (2005). Pregnancy outcome following high doses of Vitamin E supplementation. Reprod. Toxicol..

[183] Singh A., Trumpff C., Genkinger J., Davis A., Spann M., Werner E., Monk C. (2017). Micronutrient Dietary Intake in Latina Pregnant Adolescents and Its Association with Level of Depression, Stress, and Social Support. Nutrients.

